# The role of postmastectomy radiotherapy in male breast cancer: a multicenter retrospective study

**DOI:** 10.3389/fonc.2026.1801550

**Published:** 2026-05-08

**Authors:** Xi Wang, Yingli Lin, Zongjie Zheng, YongTian Chen

**Affiliations:** 1 Department of Oncological Surgery, Shaoxing Second Hospital, Shaoxing, Zhejiang, China; 2 Department of Clinical Medicine, Shaoxing University School of Medicine, Shaoxing, Zhejiang, China; 3 Department of Early Childhood Education, Shaoxing Vocational & Technical College, Shaoxing, Zhejiang, China; 4 Department of Ultrasound Medicine, Sanming First Hospital Affiliated to Fujian Medical University, Sanming, Fujian, China; 5 Department of Breast Surgery, Sanming First Hospital Affiliated to Fujian Medical University, Sanming, Fujian, China

**Keywords:** breast cancer-specific survival, male breast cancer, overall survival, postmastectomy radiotherapy, SEER database, SJTUBCDB

## Abstract

**Introduction:**

Male breast cancer (MBC) is a rare malignancy, accounting for about 1% of all breast cancers. Treatment guidelines are largely extrapolated from female breast cancer (FBC) studies, with limited MBC−specific evidence. This study evaluates the impact of postmastectomy radiotherapy (PMRT) on survival outcomes in MBC.

**Methods:**

We conducted a retrospective cohort study using the SEER database (2004–2015) and the Shanghai Jiao Tong University Breast Cancer Data Base (SJTUBCDB). Included were MBC patients with T1–2N1M0 disease after mastectomy. Inverse probability of treatment weighting (IPTW) was used to balance baseline characteristics. Overall survival (OS), breast cancer−specific survival (BCSS), and disease−free survival (DFS) were compared between PMRT and non−PMRT groups. Competing risks were accounted for in BCSS analysis using the Fine-Gray subdistribution hazard model. Subgroup analyses were based on tumor size, lymph node involvement, and hormone receptor status.

**Results:**

Of 714 eligible SEER patients, 33.9% received PMRT. PMRT was associated with improved OS (HR 0.79, 95% CI 0.53–0.94), especially in patients with T2 tumors, 2–3 positive lymph nodes, or negative progesterone receptor (PR) status. Competing risk analysis confirmed no significant difference in BCSS (subdistribution HR 0.79, 95% CI 0.44–1.42). No significant difference in BCSS was observed. In the SJTUBCDB cohort (n=50), similar directional trends were noted: PMRT was linked to better OS and DFS in high−risk subgroups, particularly in Luminal B subtypes and patients with T2 tumors or multiple involved lymph nodes.

**Conclusion:**

PMRT was associated with improved OS in selected MBC patients with high−risk features such as larger tumors or PR−negative disease. The consistent findings from SEER and SJTUBCDB support the use of PMRT in high−risk MBC patients and highlight the need for personalized treatment strategies and prospective trials.

## Introduction

Male breast cancer (MBC) is a rare condition, representing approximately 1% of all breast cancer cases worldwide (1, 2). Recent epidemiological studies indicate a rising incidence over time (3, 4). Due to its rarity, conducting randomized controlled trials to establish optimal diagnostic and treatment strategies remains challenging (5). Consequently, MBC management is largely informed by national cancer registry data, small retrospective studies, and extrapolation from clinical trials in female breast cancer (FBC).

In FBC, numerous studies have demonstrated that postmastectomy radiotherapy (PMRT) significantly improves local control and reduces mortality, especially in patients with T1–2 tumors and 1–3 positive lymph nodes (6). Although MBC shares some characteristics with FBC, important differences influence treatment. MBC tends to occur in older patients, is associated with higher rates of lymph node metastasis, and shows a greater proportion of estrogen receptor−positive (ER+) tumors (7, 8). Current guidelines recommend adjuvant radiotherapy for tumors classified as T2 or higher, hormone receptor−negative cases, or those with axillary lymph node involvement (9).

Despite these recommendations, the indications for PMRT in MBC remain uncertain. Some retrospective analyses suggest that PMRT improves survival, particularly in lymph node−positive patients (10–13), while others report no significant reduction in local recurrence or improvement in survival, even though PMRT may aid local tumor control (14, 15).

To further clarify the role of PMRT in MBC, we utilized the Surveillance, Epidemiology, and End Results (SEER) database and supplemented it with data from the Shanghai Jiao Tong University Breast Cancer Data Base (SJTUBCDB). This combined approach allowed us to investigate the association between adjuvant radiotherapy and survival outcomes across different tumor sizes, lymph node statuses, and immunohistochemical subtypes in a more robust manner. Our aim was to identify subsets of MBC patients who might safely forgo adjuvant radiotherapy and to validate findings across diverse patient cohorts.

## Methods

### Study design and data source

We searched two databases for male patients with invasive breast cancer: the Shanghai Jiao Tong University Breast Cancer Data Base (SJTUBCDB) and the National Cancer Institute’s Surveillance, Epidemiology, and End Results (SEER) database. Eligibility criteria for both cohorts included: male sex, primary invasive breast cancer, tumor size <5 cm, 1–3 positive lymph nodes, no distant metastasis, single primary carcinoma, mastectomy, and unilateral disease. We selected T1-2N1M0 patients because this group represents an intermediate-risk population in whom the benefit of PMRT remains debated, particularly in MBC where evidence is scarce. This focus allowed us to minimize stage-related heterogeneity and better isolate the treatment effect.

### Data collection

From SEER, we extracted: year of diagnosis, age, race/ethnicity, marital status, tumor size, lymph node involvement, histology, grade, ER/PR status, chemotherapy, and radiotherapy.

From SJTUBCDB, we collected: age, menopausal status, tumor stage, lymph node status, histology, grade, molecular subtype, surgery type, chemotherapy, radiotherapy, endocrine therapy, HER2−targeted therapy, and survival outcomes (DFS, OS).

### Outcomes of interest

Primary endpoints were OS, BCSS, and DFS. DFS was defined as time from surgery to recurrence (locoregional or distant). OS was defined as time from surgery to death from any cause; BCSS as time from surgery to breast cancer−specific death.

### Statistical analysis

Statistical methods employed in this study were similar to those used in previous studies examining the benefits of interventions for subsets of breast cancer patients (16, 17). Baseline characteristics between non-PMRT and PMRT groups were compared using Pearson’s Chi-square test for categorical variables and the T-test for continuous variables. To balance differences between the groups, inverse probability of treatment weighting (IPTW) was employed (18). Propensity scores were calculated using logistic regression, incorporating baseline characteristics such as age, year of diagnosis, race, marital status, histological type, nuclear grade, tumor size, number of positive lymph nodes, breast cancer subtype, and receipt of chemotherapy. Stabilized weights were used, and balance was assessed using standardized mean differences (SMD), with SMD <0.1 considered well-balanced (**Supplementary Figure 1**). After IPTW adjustment, Kaplan–Meier survival curves were utilized to estimate DFS, BCSS and OS for patients with and without PMRT across subgroups stratified by tumor stage, number of positive lymph nodes, and hormone receptor status. Multivariate Cox regression analysis was performed to identify the factors associated with survival. For BCSS, we additionally performed competing risk analysis using the Fine-Gray subdistribution hazard model, with death from other causes treated as a competing event. All statistical tests were two-sided, with significance set at p < 0.05. Analyses were conducted using R software (version 4.4.1).

## Results

### Baseline characteristics

#### SEER cohort

A total of 714 male breast cancer patients with stage T1−2N1M0 were included. Among these, 472 (66.1%) were in the non−PMRT group and 242 (33.9%) in the PMRT group. Median follow−up was 62.3 months (SD 36.72; range 1–153 months). Compared with the PMRT group, the non−PMRT group included a higher proportion of patients diagnosed in earlier years, with less lymph node involvement, and who did not receive chemotherapy. After IPTW adjustment, baseline characteristics were well balanced between the groups, with all standardized mean differences below 0.1 (**Table 1**, **Supplementary Figure 1**).

**Table 1:** Baseline characteristics of male breast cancer patients between non-PMRT and PMRT groups.

Characteristic	Unmatched		P	IPTW		P
	non-PMRT	PMRT		non-PMRT	PMRT	
	n = 472 (%)	n = 242 (%)		n = 706.1 (%)	n = 736.6 (%)	
Year of diagnosis						
2004-2007	151 (32.0)	53 (21.9)	0.001	207.8 (29.4)	235.7 (32.0)	0.82
2008-2011	164 (34.7)	75 (31.0)		238.0 (33.7)	236.1 (32.0)	
2012-2015	157 (33.3)	114 (47.1)		260.3 (36.9)	264.9 (36.0)	
Age, years						
<50	53 (11.2)	26 (10.7)	0.863	77.3 (10.9)	75.8 (10.3)	0.971
50-70	265 (56.1)	141 (58.3)		403.2 (57.1)	425.3 (57.7)	
≥70	154 (32.6)	75 (31.0)		225.7 (32.0)	235.5 (32.0)	
Marital status						
Married	326 (69.1)	154 (63.6)	0.161	477.8 (67.7)	508.6 (69.0)	0.964
Single	53 (11.2)	36 (14.9)		87.3 (12.4)	87.0 (11.8)	
Separated	64 (13.6)	42 (17.4)		101.4 (14.4)	106.1 (14.4)	
Unknown	29 (6.1)	10 (4.1)		39.7 (5.6)	34.9 (4.7)	
Race						
White	385 (81.6)	190 (78.5)	0.336	571.0 (80.9)	598.7 (81.3)	0.945
Black	57 (12.1)	40 (16.5)		93.6 (13.3)	90.1 (12.2)	
Other	27 (5.7)	10 (4.1)		37.5 (5.3)	44.6 (6.1)	
Unknown	3 (0.6)	2 (0.8)		4.0 (0.6)	3.2 (0.4)	
Grade						
I	37 (7.8)	21 (8.7)	0.327	55.6 (7.9)	50.0 (6.8)	0.945
II	268 (56.8)	120 (49.6)		384.2 (54.4)	398.3 (54.1)	
III	154 (32.6)	94 (38.8)		247.1 (35.0)	269.9 (36.6)	
Unknown	13 (2.8)	7 (2.9)		19.2 (2.7)	18.4 (2.5)	
Histology						
IDC	413 (87.5)	210 (86.8)	0.873	614.3 (87.0)	643.7 (87.4)	0.867
ILC	7 (1.5)	3 (1.2)		9.3 (1.3)	6.4 (0.9)	
Other	52 (11.0)	29 (12.0)		82.5 (11.7)	86.5 (11.7)	
T stage						
T1	229 (48.5)	105 (43.4)	0.222	327.8 (46.4)	345.7 (46.9)	0.91
T2	243 (51.5)	137 (56.6)		378.4 (53.6)	390.9 (53.1)	
Number of positive lymph nodes						
1	316 (66.9)	138 (57.0)	0.033	446.5 (63.2)	476.5 (64.7)	0.923
2	102 (21.6)	68 (28.1)		167.6 (23.7)	165.0 (22.4)	
3	54 (11.4)	36 (14.9)		92.0 (13.0)	95.1 (12.9)	
ER status						
Negative	13 (2.8)	6 (2.5)	0.079	16.6 (2.3)	11.2 (1.5)	0.741
Positive	435 (92.2)	232 (95.9)		661.6 (93.7)	699.7 (95.0)	
Unknown	24 (5.1)	4 (1.7)		28.0 (4.0)	25.7 (3.5)	
PR status						
Negative	56 (11.9)	24 (9.9)	0.096	77.5 (11.0)	71.4 (9.7)	0.863
Positive	389 (82.4)	212 (87.6)		595.8 (84.4)	635.1 (86.2)	
Unknown	27 (5.7)	6 (2.5)		32.8 (4.6)	30.1 (4.1)	
Chemotherapy						
No	239 (50.6)	76 (31.4)	<0.001	314.2 (44.5)	345.2 (46.9)	0.605
Yes	233 (49.4)	166 (68.6)		392.0 (55.5)	391.4 (53.1)	

#### SJTUBCDB cohort

From the institutional database, 50 eligible MBC patients were identified: 16 (32.0%) in the non−PMRT group and 34 (68.0%) in the PMRT group. Median follow−up was 49.6 months (SD 43.21; range 1–176 months). The PMRT group was significantly younger, with a higher proportion of patients aged 50–70 years (79.4% vs. 31.2%, p<0.05), and nearly all (91.2%) received adjuvant endocrine therapy compared with 56.2% in the non−PMRT group (p=0.012). The distribution of histology, grade, T stage, nodal involvement, molecular subtypes, and receipt of chemotherapy was comparable between the groups (**Supplementary Table 1**).

### Survival analysis for all patients

#### SEER cohort

**Figure 1:**
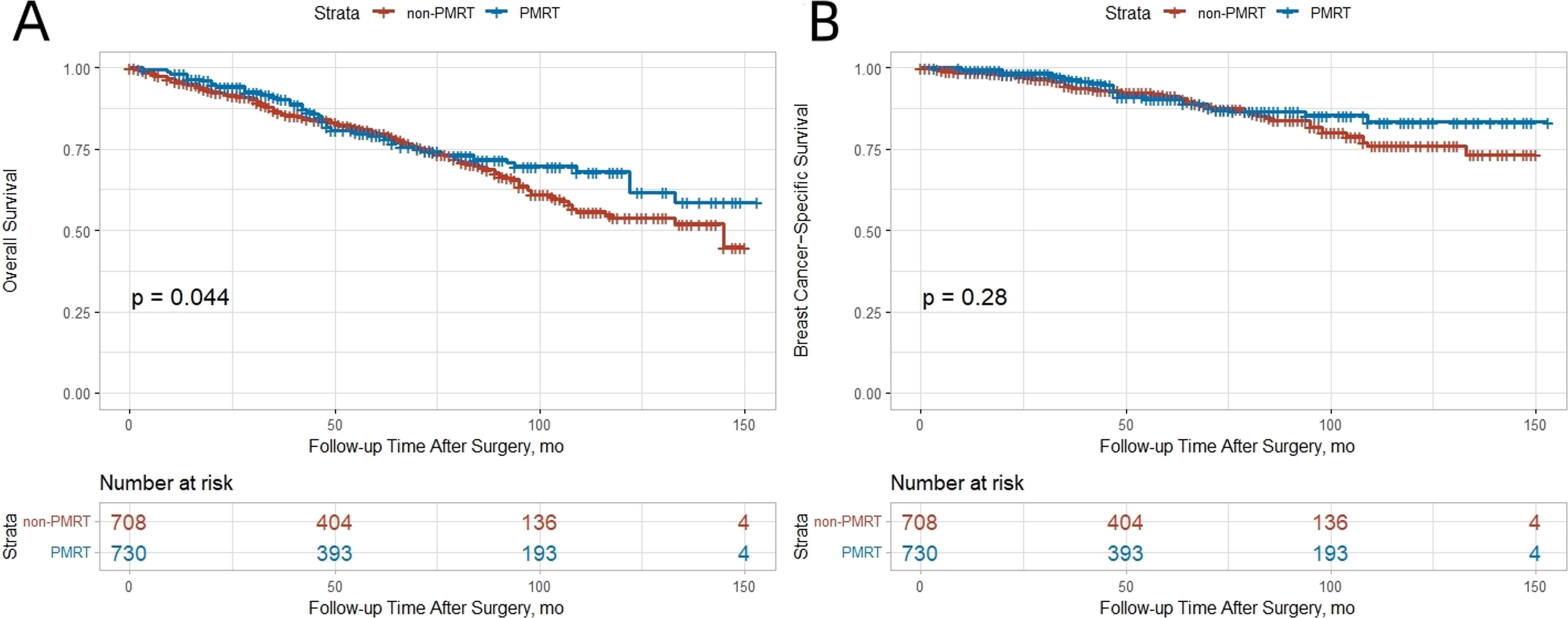
Kaplan-Meier curves comparing outcomes between non-PMRT and PMRT groups after IPTW adjustment. **(A)** OS; **(B)** BCSS.

There were 77 breast cancer−related deaths (10.8%) and 106 deaths from other causes (14.8%). After IPTW adjustment, OS was significantly better in the PMRT group (**Figure 1A**). The 10−year OS rate was 68.0% in the PMRT group versus 53.8% in the non−PMRT group (absolute benefit 14.2%). No significant difference in BCSS was observed between the groups (**Figure 1B**). In competing risk analysis, no significant difference in BCSS was observed between the groups (subdistribution HR 0.79, 95% CI 0.44–1.42), consistent with the original Cox regression findings. The 10−year BCSS rate was 83.3% with PMRT and 75.9% without.

#### SJTUBCDB cohort

**Figure 2:**
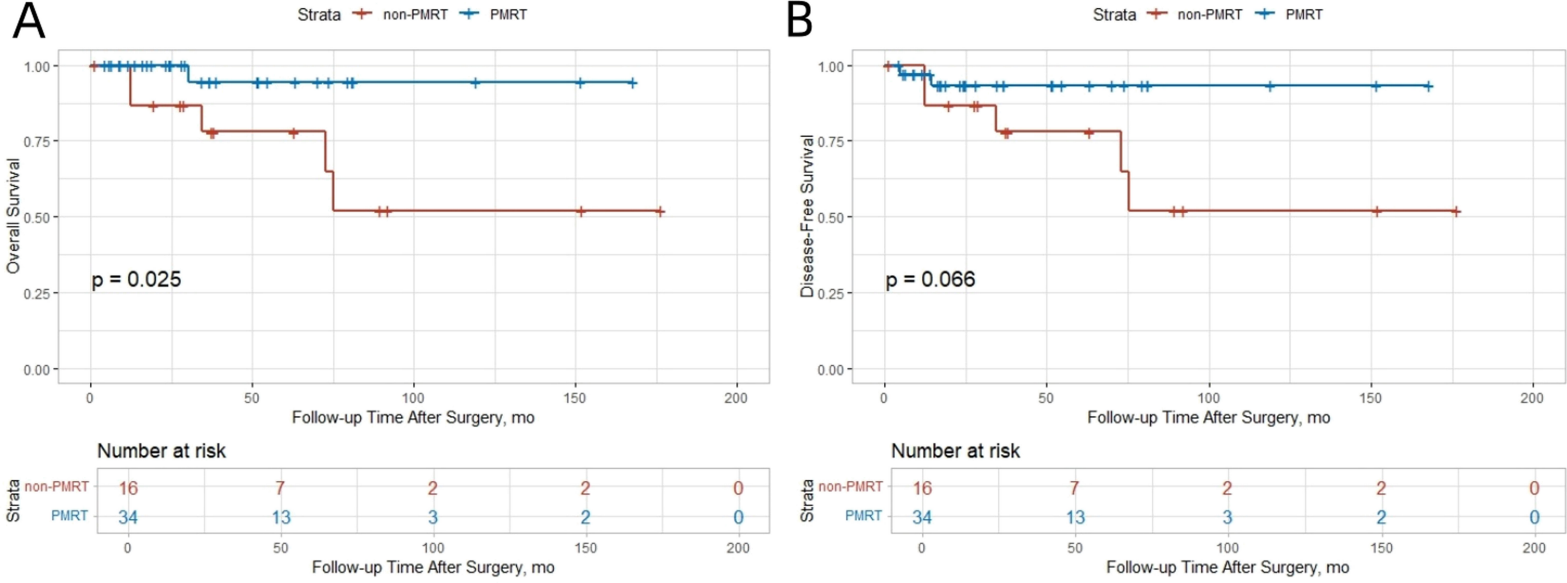
Kaplan-Meier curves for overall survival and disease-free survival in the SJTUBCDB cohort. **(A)** OS; **(B)** DFS.

There were 5 deaths (10%) and 6 recurrences (12%). Consistent with SEER findings, the PMRT group showed a trend toward improved survival. The 5−year OS rate was 52.0% in the non−PMRT group and 94.4% in the PMRT group (**Figure 2A**). DFS was notably better in the PMRT group, with a 5−year DFS rate of 93.2% compared with 52.0% in the non−PMRT group (**Figure 2B**).

### Survival analysis stratified by clinical characteristics

#### T stage

**Figure 3:**
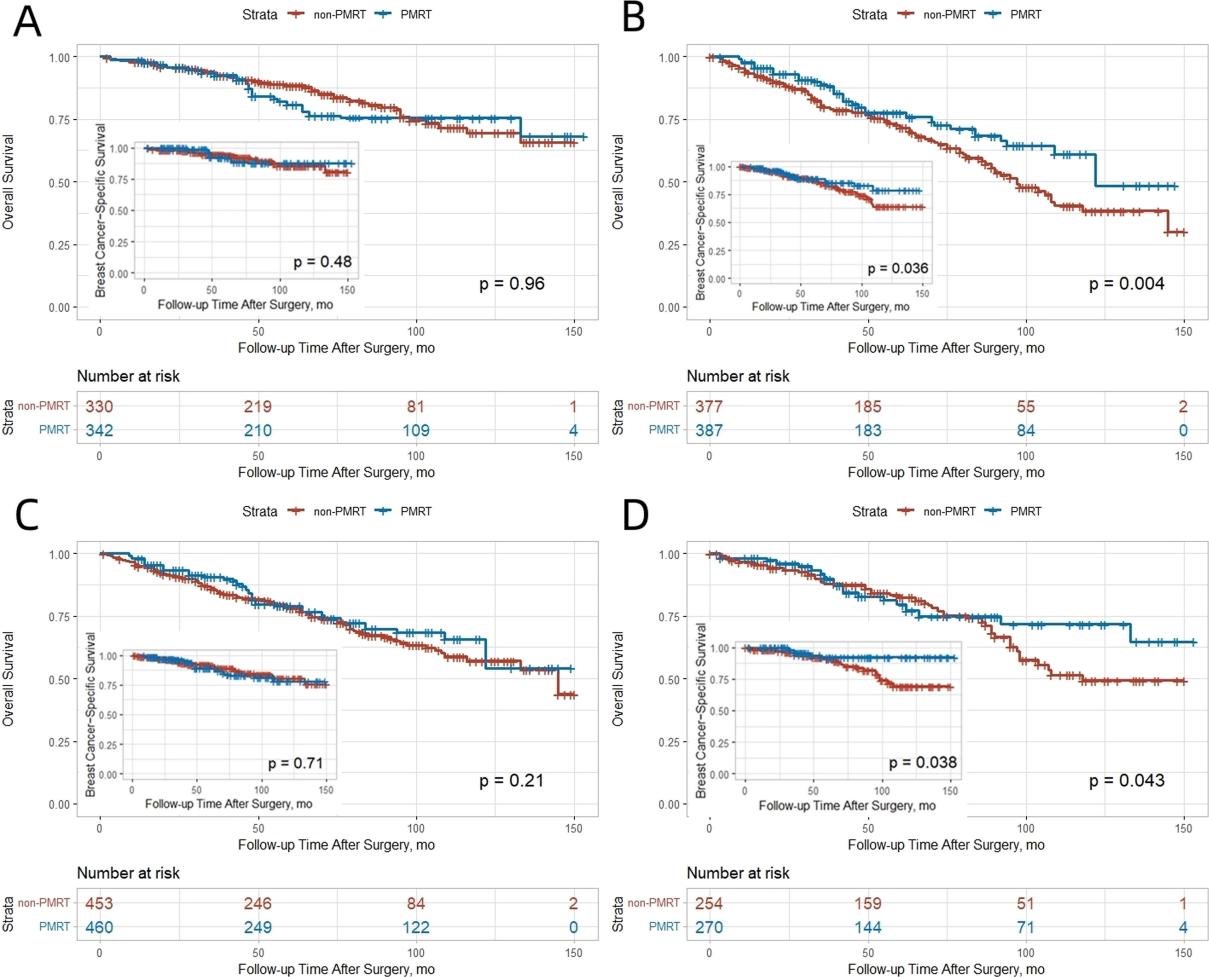
Kaplan-Meier curves for OS and BCSS stratified by tumor stage and lymph node involvement in the SEER cohort after IPTW adjustment. **(A)** T1 Stage. **(B)** T2 Stage. **(C)** 1 Positive Lymph Node. **(D)** 2–3 Positive Lymph Nodes.

**Figure 4:**
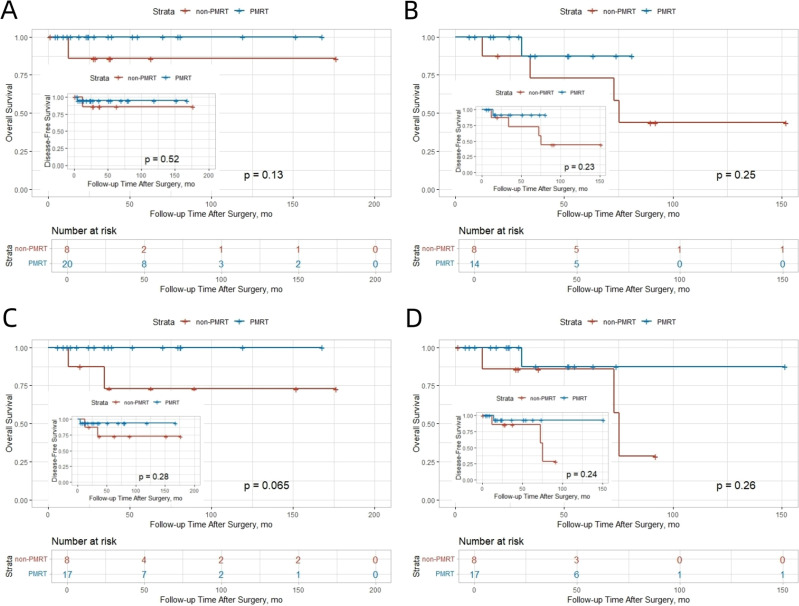
Kaplan-Meier curves for OS and DFS stratified by tumor stage and lymph node involvement in the SJTUBCDB cohort. **(A)** T1 Stage. **(B)** T2 Stage. **(C)** 1 Positive Lymph Node. **(D)** 2–3 Positive Lymph Nodes.

In the SEER cohort, PMRT significantly improved both OS and BCSS in patients with T2 tumors. The 10−year OS and BCSS rates were 38.4% and 64.1% in the non−PMRT group, compared with 56.8% and 78.7% in the PMRT group (absolute benefits 18.4% and 14.6%, respectively). No significant benefit was observed for T1 tumors (**Figures 3A, B**). This differential pattern was corroborated in the SJTUBCDB cohort, where non−significant trends toward improved DFS and OS were observed in both T1 and T2 patients receiving PMRT (**Figures 4A, B**).

### Number of positive lymph nodes

In the SEER cohort, patients with 2–3 positive lymph nodes derived significant OS and BCSS benefit from PMRT (OS: HR 0.51, 95% CI 0.34–0.77; BCSS: HR 0.45, 95% CI 0.24–0.83), whereas those with only 1 positive node did not (**Figures 3C, D**). The 10−year OS and BCSS rates were 71.6% and 92.5% in the PMRT group versus 49.2% and 69.3% in the non−PMRT group (absolute benefits 22.4% and 23.2%). A similar trend was observed in the SJTUBCDB cohort, where PMRT was associated with a non−significant improvement in DFS for patients with either 1 or 2–3 positive nodes (**Figures 4C, D**).

### Hormone receptor status/molecular subtype

**Figure 5:**
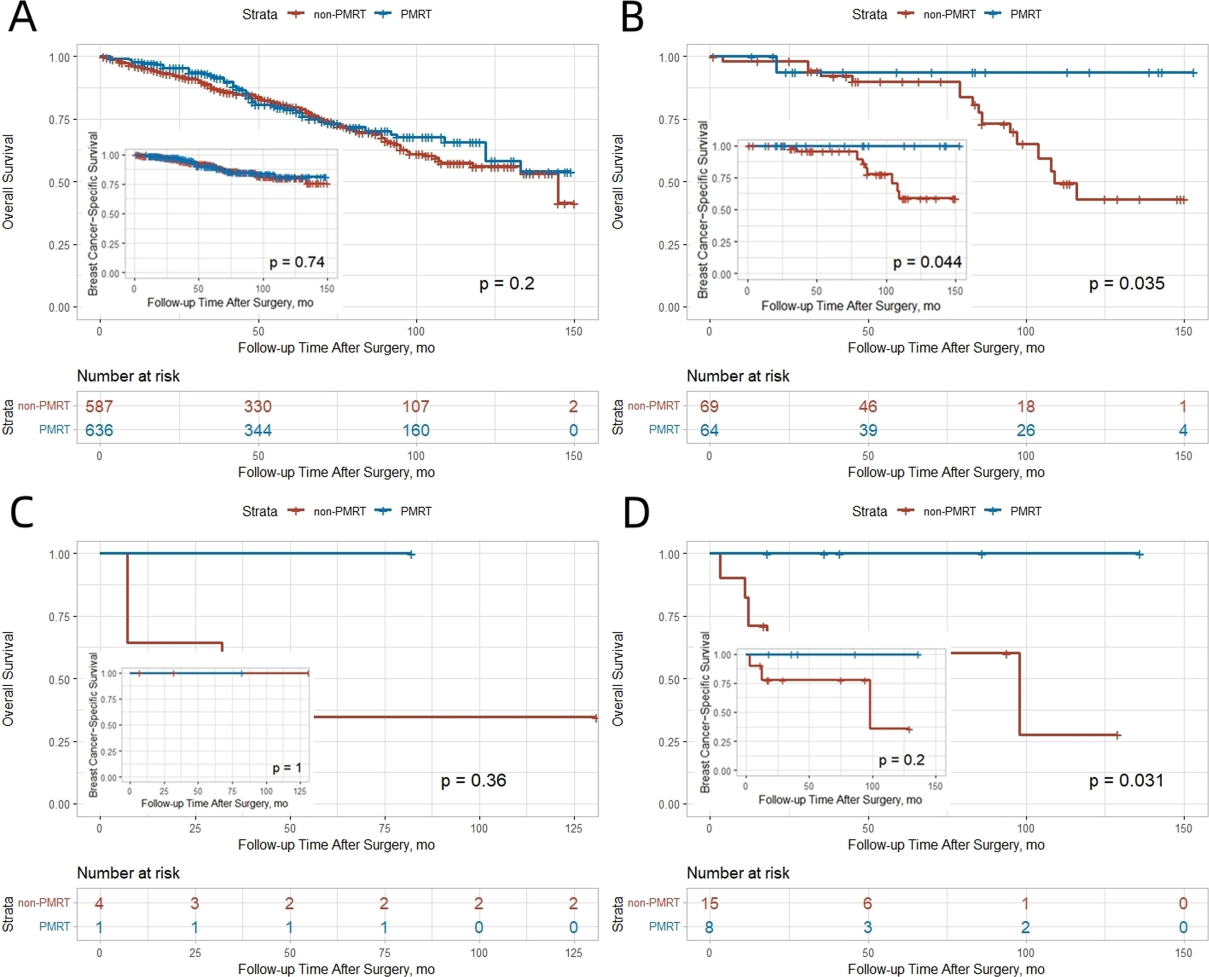
Kaplan-Meier curves for OS and BCSS stratified by hormone receptor status in the SEER cohort after IPTW adjustment. **(A)** ER+/PR+. **(B)** ER+/PR-. **(C)** ER-/PR+. **(D)** ER-/PR-.

In the SEER cohort, no significant OS or BCSS differences were noted in PR−positive patients. However, in ER−positive/PR−negative patients, PMRT showed significantly better OS and BCSS (10−year OS 93.7% vs. 43.1%; BCSS 100% vs. 58.9%; absolute benefits 50.6% and 41.1%). In ER−negative/PR−negative patients, PMRT significantly improved 10−year OS (100% vs. 27.7%) but not BCSS (**Figure 5**).

**Figure 6:**
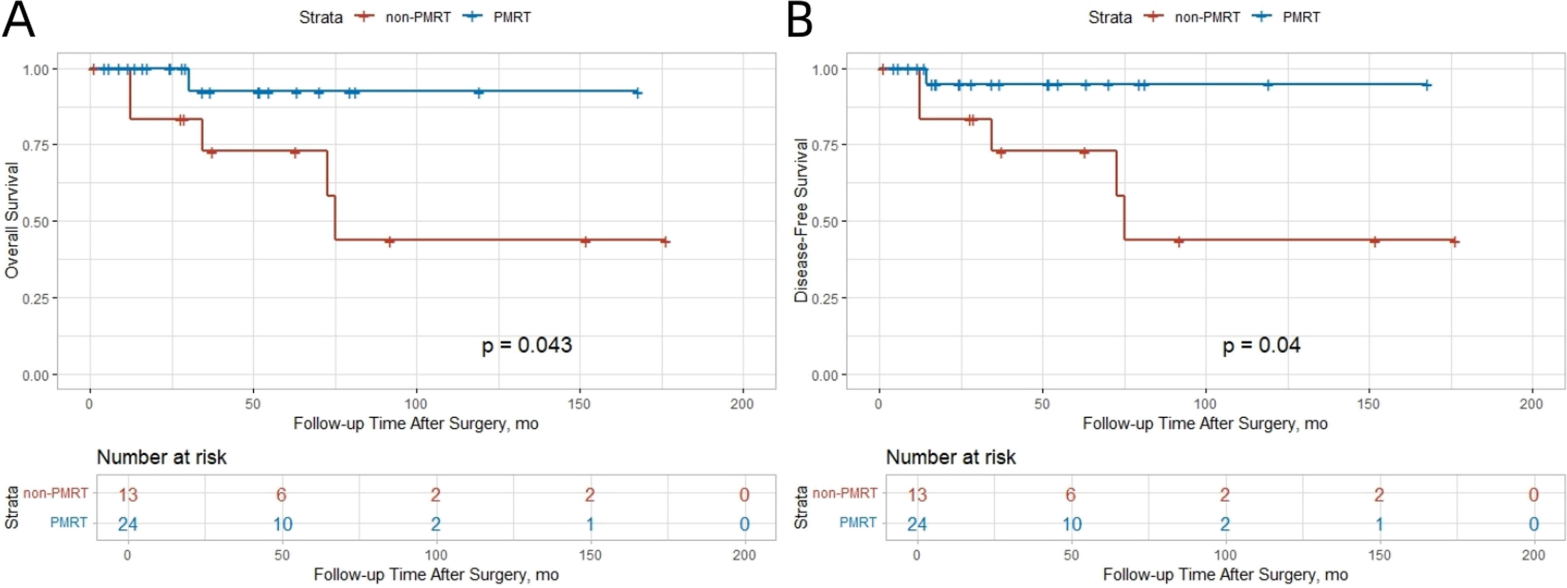
Kaplan-Meier Curves for Overall Survival and Disease-Free Survival in Luminal B Subtype Patients from the SJTUBCDB Cohort. **(A)** OS; **(B)** DFS.

Analysis of the SJTUBCDB cohort, which classified patients by integrated molecular subtype, provided complementary insights. Specifically within the Luminal B subtype (typically ER−positive, with variable PR expression and higher proliferation), PMRT was associated with a consistent trend toward improved survival. Kaplan−Meier curves suggested a clinical benefit in both OS and DFS for Luminal B patients receiving PMRT (**Figure 6**). This observation aligns directionally with the SEER finding of particular PMRT benefit in the ER−positive/PR−negative subgroup, a phenotype often encompassed within the Luminal B classification.

### Multivariate analysis of the unmatched cohort

Multivariate analysis identified PMRT (HR 0.79, 95% CI 0.53–0.94) and chemotherapy (HR 0.59, 95% CI 0.40–0.87) as independent predictors of improved OS. Although both PMRT and chemotherapy showed a trend toward better BCSS, neither reached statistical significance. Later year of diagnosis was also associated with improved OS (HR 0.45, 95% CI 0.23–0.89). Conversely, larger tumor size (T2) (OS: HR 2.07, 95% CI 1.38–3.10; BCSS: HR 1.90, 95% CI 1.23–3.62), age >70 years (HR 3.81, 95% CI 1.72–8.46), and divorced/widowed marital status (HR 1.85, 95% CI 1.27–2.71) were associated with worse survival outcomes (**Table 2**).

**Table 2:** Multivariate analysis of prognostic factors influencing BCSS and OS in male breast cancer patients.

Characteristic	BCSS		P	OS		P
	HR	95%CI		HR	95%CI	
Year of diagnosis						
2004-2007	Reference			Reference		
2008-2011	0.98	0.51 ~ 1.88	0.945	1.02	0.67 ~ 1.55	0.917
2012-2015	0.57	0.22 ~ 1.50	0.256	0.45	0.23 ~ 0.89	0.022
Age, years						
<50	Reference			Reference		
50-70	0.87	0.39 - 1.96	0.745	1.55	0.72 - 3.36	0.262
≥70	2.01	0.87 - 4.63	0.103	3.81	1.72 - 8.46	0.001
Marital status						
Married	Reference			Reference		
Single	0.96	0.44 - 2.11	0.926	1.33	0.85 - 2.08	0.208
Separated	2.35	1.05 - 5.30	0.039	1.85	1.27 - 2.71	0.001
Unknown	1.09	0.36 - 3.29	0.872	1.23	0.61 - 2.48	0.564
Race						
White	Reference			Reference		
Black	2.39	1.37 - 4.15	0.002	1.69	1.07 - 2.66	0.023
Other	1.62	0.55 - 4.76	0.382	0.68	0.29 - 1.62	0.388
Grade						
I	Reference			Reference		
II	2.35	0.75 - 7.34	0.143	1.33	0.68 - 2.61	0.411
III	3.28	1.00 - 10.77	0.050	1.81	0.92 - 3.51	0.081
T stage						
T1	Reference			Reference		
T2	1.90	1.23 - 3.62	0.041	2.07	1.38 - 3.10	<0.001
Number of positive lymph nodes						
1	Reference			Reference		
2	0.72	0.33 - 1.55	0.395	0.93	0.58 - 1.51	0.780
3	1.31	0.65 - 2.64	0.456	1.09	0.65 - 1.83	0.703
ER status						
Negative	Reference			Reference		
Positive	0.85	0.16 - 4.51	0.850	0.49	0.19 - 1.26	0.138
PR status						
Negative	Reference			Reference		
Positive	0.83	0.40 - 1.73	0.627	1.12	0.61 - 2.07	0.709
Chemotherapy						
No	Reference			Reference		
Yes	0.75	0.46 - 1.22	0.239	0.59	0.40 - 0.87	0.007
PMRT						
No	Reference			Reference		
Yes	0.77	0.43 ~ 1.37	0.368	0.79	0.53 ~ 0.94	0.034

## Discussion

The optimal use of postmastectomy radiotherapy (PMRT) in male breast cancer (MBC) remains poorly defined, largely due to the rarity of the disease and the consequent lack of randomized evidence (19). Although treatment paradigms are often extrapolated from female breast cancer (FBC), emerging data highlight distinct clinicopathological and biological differences between the sexes, underscoring the need for gender−specific guidelines (20–24). Our study leverages complementary data from the population−based SEER registry and the clinically detailed SJTUBCDB to provide a nuanced analysis of PMRT benefit in T1−2N1M0 MBC.

MBC typically occurs after age 50 and has a higher prevalence of hormone receptor positivity compared with FBC. These clinicopathological and genetic differences indicate that MBC and FBC are distinct diseases (20–24). Although evidence exists for the benefit of endocrine and targeted therapies in male patients, the impact of PMRT remains unclear (25–27). Therefore, directly applying PMRT strategies established for FBC to MBC may not be appropriate. In fact, men tend to receive PMRT less frequently than women, likely due to uncertainties about its efficacy (28). Previous studies have yielded mixed results: some retrospective analyses reported improved local control with PMRT but no overall survival benefit (29, 30), while others found no survival advantage (10). Conversely, several studies have shown that PMRT improves both local control and survival, particularly in early and locally advanced MBC with positive nodes or stage III disease (12, 13, 31, 32). These conflicting findings highlight the complexity of determining the role of PMRT in MBC.

In fact, the true impact of adjuvant PMRT on disease-free survival (DFS) and OS in MBC patients has been difficult to assess due to limited statistical power in most studies. Based on retrospective data, establishing clear indications for PMRT in MBC remains challenging. Our analysis, which included patients diagnosed between 2004 and 2015 with a follow-up period exceeding five years, utilized IPTW to enhance the reliability of the findings. We observed that PMRT was significantly associated with improved OS in MBC patients, particularly among those with larger tumors (T2), two to three positive lymph nodes, or negative progesterone receptor (PR) status.

However, the improvement in OS without a corresponding statistically significant benefit in BCSS should be interpreted cautiously. Several factors may explain this discrepancy. First, male breast cancer patients are often older and have competing comorbidities; non-breast cancer deaths, particularly cardiovascular events, account for a substantial proportion of mortality in this population, as demonstrated in a large SEER-based analysis of male breast cancer patients (33). Second, BCSS events were fewer in number, limiting statistical power. The competing risk analysis confirmed the lack of a significant BCSS difference, supporting the robustness of this finding.

The integration of the SJTUBCDB institutional cohort enriches this analysis by providing a clinically detailed, though smaller, validation set. Given the small sample size and baseline imbalances (e.g., younger age and higher endocrine therapy use in the PMRT group), the SJTUBCDB cohort should be viewed as providing supportive, exploratory evidence rather than independent confirmation of the SEER findings. While the limited sample size precluded statistically significant findings in its subgroup analyses, the directional trends are instructive and consistent with the SEER results. In the SEER database, subgroup analysis indicated limited PMRT benefit in PR−positive patients, likely due to the efficacy of modern systemic therapies. Correspondingly, in the SJTUBCDB cohort, the trend toward improved outcomes with PMRT in Luminal B subtype patients aligns biologically with the SEER finding of significant benefit in the ER−positive/PR−negative subgroup, as PR−low or negative status is a hallmark of many Luminal B tumors. It is important to note that ER+/PR- status is not strictly equivalent to the integrated molecular classification of Luminal B; therefore, the observed alignment is directional and hypothesis-generating rather than confirmatory. This concordance across two methodologically different datasets strengthens the hypothesis that a true clinical benefit exists in these high−risk groups, which may be underpowered for detection in smaller single−institution studies.

This study has limitations inherent to its retrospective design, including potential selection biases. Key unmeasured confounders, such as comorbidities (e.g., cardiovascular disease, diabetes), family history, margin status, lymphovascular invasion, detailed systemic therapy (e.g., specific chemotherapy regimens, duration of endocrine therapy), and radiation dose/fractionation, were not available in the SEER database and may have influenced outcomes. Additionally, the influence of irradiation dose and fractionation on outcomes could not be evaluated, as these details are also not captured by SEER. The small size of the SJTUBCDB cohort further limits definitive conclusions from its data and precluded the application of advanced statistical adjustments like IPTW that were possible in the larger SEER cohort. Moreover, the performance of multiple subgroup analyses increases the risk of type I error; therefore, these findings should be considered hypothesis-generating rather than confirmatory. Consequently, while subgroup analyses were conducted to explore the benefits of radiotherapy in patients with controversial indications, residual confounding in both datasets may still obscure the true benefits of PMRT. To address these gaps, a prospective randomized trial investigating PMRT in MBC is urgently needed.

## Conclusion

In conclusion, this dual-database analysis provides compelling real-world evidence that PMRT is associated with improved survival in MBC patients with high-risk clinicopathological features, including T2 stage, multiple involved lymph nodes, and PR-negative disease. The consistent directional trends in the SJTUBCDB cohort, despite its size limitations, lend supportive credibility to the significant associations found in the SEER data. While our findings support the use of PMRT in these high-risk groups, further prospective studies are needed to confirm its benefits and guide treatment strategies for MBC.

## Data Availability

The original contributions presented in the study are included in the article/**Supplementary Material**. Further inquiries can be directed to the corresponding author.
