# Supplementary figures and images for: The role of postmastectomy radiotherapy in male breast cancer: a multicenter retrospective study
Source: Front Oncol. 2026 May 8;16:1801550. doi: 10.3389/fonc.2026.1801550 (PMC13193992; doi:10.3389/fonc.2026.1801550)

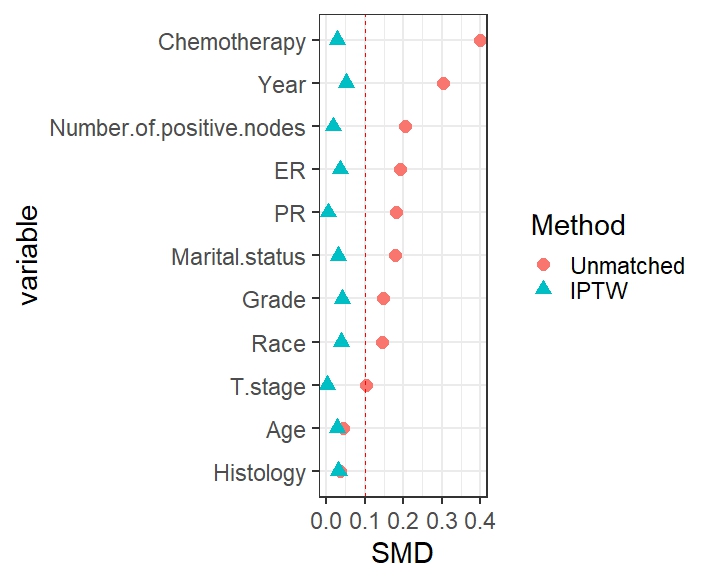

Supplement: Supplementary file 1 [file Image1.jpeg]
