# Supplementary material for: The role of postmastectomy radiotherapy in male breast cancer: a multicenter retrospective study
Source: Front Oncol. 2026 May 8;16:1801550. doi: 10.3389/fonc.2026.1801550 (PMC13193992; doi:10.3389/fonc.2026.1801550)
Supplement: Supplementary file 2 [file Table1.docx]

Supplement Table 1. Baseline Characteristics of Male Breast Cancer Patients Between Non-PMRT and PMRT Groups in SJTUBCDB

| Characteristic | non-PMRT | PMRT | P |
| --- | --- | --- | --- |
|  | n = 16 (%) | n = 34 (%) |  |
| Age, years |  |  |  |
| ＜50 | 1 (6.2) | 3 (8.8) | <0.05 |
| 50-70 | 5 (31.2) | 27 (79.4) |  |
| ≥70 | 10 (62.5) | 4 (11.8) |  |
| Histology |  |  |  |
| IDC | 15 (93.8) | 31 (91.2) | 0.786 |
| ILC | 0 (0.0) | 1 (2.9) |  |
| Other | 1 (6.2) | 2 (5.9) |  |
| Grade |  |  |  |
| I | 0 (0.0) | 1 (4.2) | 0.573 |
| II | 11 (84.6) | 17 (70.8) |  |
| III | 2 (15.4) | 6 (25.0) |  |
| T stage |  |  |  |
| T1 | 8 (50.0) | 20 (58.8) | 0.779 |
| T2 | 8 (50.0) | 14 (41.2) |  |
| Number of positive lymph nodes | | | |
| 1 | 8 (50.0) | 17 (50.0) | 0.417 |
| 2 | 4 (25.0) | 13 (38.2) |  |
| 3 | 4 (25.0) | 4 (11.8) |  |
| Molecular subtype |  |  |  |
| Luminal A | 3 (23.1) | 4 (14.3) | 0.419 |
| Luminal B | 10 (76.9) | 19 (67.9) |  |
| HER2-overexpressing | 0 (0.0) | 3 (10.7) |  |
| TNBC | 0 (0.0) | 2 (7.1) |  |
| Surgery |  |  |  |
| SLNB | 2 (14.3) | 10 (30.3) | 0.432 |
| ALND | 12 (85.7) | 23 (69.7) |  |
| Chemotherapy |  |  |  |
| No | 6 (37.5) | 11 (32.4) | 0.969 |
| Yes | 10 (62.5) | 23 (67.6) |  |
| HER2-Targeted therapy |  |  |  |
| No | 16 (100.0) | 30 (88.2) | 0.383 |
| Yes | 0 (0.0) | 4 (11.8) |  |
| Endocrine therapy |  |  |  |
| No | 7 (43.8) | 3 (8.8) | 0.012 |
| Yes | 9 (56.2) | 31 (91.2) |  |
